# Exclusive vital labeling of myonuclei for studying myonuclear arrangement in mouse skeletal muscle tissue

**DOI:** 10.1186/s13395-020-00233-6

**Published:** 2020-05-07

**Authors:** Robert Louis Hastings, Ryan T. Massopust, Seth G. Haddix, Young il Lee, Wesley J. Thompson

**Affiliations:** 1 Texas A&M Institute for Neuroscience, College Station, TX USA; 2 Department of Biology, Texas A&M University, College Station, TX USA

**Keywords:** *mdx*, Central nuclei, Histone H2B, HSA, Human skeletal actin, RG, Soleplate nuclei

## Abstract

**Background:**

The arrangement of myonuclei in skeletal muscle tissue has long been used as a biomarker for muscle health, but there is a dearth of in vivo exploration of potential effects of myonuclear organization on the function and regeneration of skeletal muscle because traditional nuclear stains are performed on postmortem tissue. Therefore, we sought a transgenic method to produce a selective and persistent myonuclear label in whole muscles of living mice.

**Methods:**

We bred together a mouse line with skeletal muscle fiber-selective expression of Cre recombinase and a second mouse line with a Cre-inducible fluorescently tagged histone protein to generate a mouse line that produces a myonuclear label suitable for vital imaging and histology of fixed tissue. We tested the effectiveness of this vital label in three conditions known to generate abnormal myonuclear positioning. First, we injured myofibers of young mice with cardiotoxin. Second, this nuclear label was bred into a murine model of Duchenne muscular dystrophy. Finally, we examined old mice from this line that have undergone the natural aging process. Welch’s *t* test was used to compare wild type and transgenic mice.

**Results:**

The resulting mouse line transgenically produces a vital red fluorescent label of myonuclei, which facilitates their in vivo imaging in skeletal muscle tissue. Transgenic fluorescent labeling of myonuclei has no significant effect on skeletal muscle function, as determined by twitch and tetanic force recordings. In each muscle examined, including those under damaged, dystrophic, and aged conditions, the labeled myonuclei exhibit morphology consistent with established literature, and reveal a specialized arrangement of subsynaptic myonuclei at the neuromuscular junction.

**Conclusions:**

Taken together, our results demonstrate that this mouse line provides a versatile tool to selectively visualize myonuclei within both living and fixed preparations of healthy, injured, diseased, and aged muscles.

## Background

A skeletal muscle is composed of a variety of cell types, such as myofibers, resident macrophages, muscle stem cells (satellite cells), and Schwann cells. A complete understanding of how skeletal muscle reacts to injury and disease requires the study of the interaction between these various cell types in the system as a whole. Skeletal myofibers, the focus of this study, are multinucleate syncytia. Myonuclei in a healthy, undamaged adult myofiber lie along the periphery of the myofiber, just under the sarcolemma [1]. The relationship between muscle function and the arrangement, size, and activity of myonuclei is an understudied field, though recent in vivo experiments in fruit flies have described a relationship between nuclear scaling and muscle cell size and function [2]. In mice, myonuclei have largely been relegated to the role of a biomarker for stages of development and regeneration. Muscle fiber damage followed by regeneration of the myofiber results in the appearance of “central nuclei,” or myonuclei in the approximate center of the myofiber, rather than the periphery [3]. These central nuclei appear in skeletal muscle fibers after deliberate muscle damage induced by a variety of insults, including myotoxic drug exposure, muscle transection, laser ablation, and eccentric contractions [4–10]. As such, central nuclei have long been accepted as a morphological indicator for muscle health, specifically for myofibers having undergone a degeneration/regeneration event. Central nuclei are also a prominent feature of aged and myopathic skeletal muscle [11–16], suggesting that aging and myopathic muscle fibers are undergoing increased bouts of regeneration/degeneration. Therefore, a better understanding of myonuclear disposition and dynamics may be informative about the aging, disease, and injury of skeletal muscle tissue.

Myonuclei are difficult to study using traditional nuclear stains such as DAPI and Hoechst because they indiscriminately stain all nuclei in the tissue. It is difficult to identify in whole mounted muscle tissue which of these stained nuclei reside within the myofibers because skeletal muscle tissue contains the nuclei of a variety of cell types and many of these cells are pressed tightly against the sarcolemma. Furthermore, although Hoechst is sometimes used for live staining in vitro [17, 18], it is not ideal as a vital stain because staining through the entire depth and along the entire length of the muscle in vivo would likely require an impractical concentration and/or incubation period. Therefore, traditional nuclear stains are of limited use in tracking myonuclei over time. We sought a transgenic strategy to fluorescently, and stably, label myonuclei in living mice. Here we characterize a mouse line, henceforth referred as the “RG” mice, in which myonuclei are labeled with the fluorescent protein mCherry. These mice are a significant improvement on currently available technology to image myonuclei in vivo because they produce a vital, stable fluorescent label exclusive to myonuclei that can also be imaged in chemically fixed post-mortem tissue.

## Methods

### Animals

All experimental procedures were approved by Texas A&M University Institutional Animal Care and Use Committee and conducted in accordance with the National Institutes of Health guidelines. Animals were sacrificed via intraperitoneal injection of Euthanasia-III Solution (Med-Pharmex).

The “RG” mice were generated by crossing two lines of genetically modified mice, both on a C57BL/6 background. Both male and female, primarily young adults (2-6 months old), mice were used in this study. Aged mice in this study are defined as being older than 18 months. The first mouse line possesses a transgene, the “HSA-Cre79” allele (Jackson Lab #006149), that drives Cre recombinase under the control of the human α-skeletal actin promoter, which begins expression on embryonic day 9 [19]. The second mouse line possesses a Cre-inducible fluorescent reporter construct, the “H2B/GPI” allele (RIKEN CDB0227K, http://www.clst.riken.jp/arg/reporter_mice.html), knocked into the Rosa26 locus and driven by the Rosa26 promoter [20]. This reporter contains a floxed triply repeated SV40 polyadenylation sequence in front of an open reading frame encoding mCherry-tagged histone H2B protein and enhanced green fluorescent protein (GFP) fused to a glycosylphosphatidylinositol (GPI) membrane insertion signal sequence, separated by a self-cleavage 2A peptide sequence. Mice carrying the HSA-Cre79 and H2B/GPI alleles produced by breeding, referred to with the acronym “RG” (red nucleus, green plasma membrane) in accordance with the original manuscript characterizing this reporter construct [21], have skeletal muscle fibers in which the myonuclei are labeled with mCherry, and GFP is inserted into the sarcolemma. Except where otherwise indicated, all mice used for figures in this publication were heterozygous for the H2B/GPI allele and no obvious differences in fluorescent intensity have been noted between male and female RG mice, nor between mice that were hetero/homozygous for the reporter.

The RG mice were also crossed with *mdx* mice (Jackson Lab #001801), the most common murine model of Duchenne muscular dystrophy [22]. The *mdx* mice were bred on a C57BL/10 background, and therefore the “RG-*mdx*” mice produced by this cross possess three mutant alleles (HSA-Cre, H2B/GPI, and *mdx*) and have a mixed background of C57BL/6 and C57BL/10. Only male RG-*mdx* mice were used in these studies because, as with Duchenne muscular dystrophy, mdx-associated muscular dystrophy is an X-linked disease.

### Histology

Animals were sacrificed, transcardially perfused with phosphate buffered saline (PBS), and the muscles were immediately dissected, pinned in a dish, and fixed in 4% paraformaldehyde (PFA) in PBS for 20 min. The muscles were then stained and prepared for either whole mount or cross-sections. Whole-mount preparations were made by pinning the whole muscle into a sylgard-lined dish and using microsnips and forceps to peel the top layer of muscle fibers away from the surface of the muscle. The resulting “fillets” were mounted on a slide and coverslipped. Cross-sections of muscles were made by imbedding the muscle in Tissue-Tek Optimal Cutting Temperature compound (Sakura Finetek, Torrance, CA) and freezing the block for 10 s in liquid N_2_-cooled isopentane at a temperature of − 80 °C. The tissue was then sectioned on a Leica CM 3050S cryostat at 20 μm thickness. Sections were stained with mouse anti-dystrophin primary antibody (Leica Biosystems, NCL-DYS2) followed by goat anti-mouse IgG1 secondary antibody conjugated to Alexa Fluor 647 (Life Technologies A-21240) and DAPI. Fixed tissue was imaged using a Leica DMRX epifluorescence microscope, a Zeiss LSM 780 confocal microscope at the Texas A&M College of Veterinary Medicine & Biomedical Sciences Image Analysis Lab, or a Leica S5 confocal microscope at the University of Texas at Austin.

### Single fiber isolation

To isolate single myofibers, extensor digitorum longus (EDL) muscles were dissected from the mouse and incubated in 1 μg/mL BTX-555 (Alexa Fluor 555-tagged α-bungarotoxin, Invitrogen B35451) dissolved in a calcium chelating solution, called “relaxing solution,” (137 mM NaCl, 5.4 mM KCl, 5 mM MgCl2, 4 mM EGTA, 5 mM HEPES, pH 7.0) for 15 min and then washed in relaxing solution, which was made according to a previously published protocol [23]. The muscles were then fixed in 4% PFA in PBS, dissolved in relaxing solution for 1 h, and washed in a relaxing solution. The isolation process eliminated the fluorescent signal of the mCherry proteins, so an anti-mCherry antibody was used to recover the myonuclear label. The muscles were incubated in standard blocking solution (0.3% Triton X-100, 0.2% bovine serum albumin, 0.1% sodium azide) for 30 min, followed by incubation in primary antibody (goat anti-mCherry, Sicgen AB0040-200) overnight at 4 °C. The next morning, the tissue was washed in relaxing solution, fixed again in 4% PFA for 30 min, washed again in relaxing solution, incubated in 40% NaOH for 1 h, and washed in relaxing solution. The relaxing solution washes to this point in the procedure all consisted of a 5-min wash repeated 3 times. Next, the tissue was incubated with 0.5 μg/mL DAPI and 2 μg/mL BTX-555 dissolved in relaxing solution for 30 min and washed once with relaxing solution for 15 min. The secondary antibody (rabbit anti-goat Alexa Fluor 647, Invitrogen A-21446) was added to the dish for 2 h and afterwards washed once for 15 min. Forceps and a dissecting microscope were used to mount individual muscle fibers onto slides in an anti-fade fluorescence mounting medium.

### Force recordings

C57BL/6 mice, purchased from The Jackson Laboratories (Maine, USA), and RG mice were 2 to 3 months old when sacrificed for physiological recordings. Following euthanasia, animals were weighed and the soleus muscle was removed from tendon to tendon with the soleus nerve intact. Soleus (SOL) muscles were dissected with the proximal tendon attached to a piece of bone to provide an anchor which was pinned to a Sylgard dish. A hook force transducer (Harvard Apparatus, model 60-2996) was then attached through the distal tendon. Muscles were perfused/superfused with oxygenated Ringer’s solution during dissection/force recordings, respectively. The optimum length of a muscle was determined by delivering suprathreshold pulses to the nerve and stretching the muscle until no increases in force were noted. Isometric twitch forces were generated via a single suprathreshold (10-20 V) pulse using a Grass stimulator (Grass Instruments, model S88B). Direct stimulation (150 V) was delivered to the muscle through two titanium plates surrounding the muscle near its midsection. Indirect stimulation was delivered to the nerve via a suction electrode into which the attached nerve was inserted along with oxygenated Ringer’s. Tetanic force was generated by delivering a 60 Hz stimulation for 2 s. As with twitch responses, direct and indirect stimulations were applied.

The twitch and tetanic force recordings were displayed on a digital oscilloscope (Tektronics, model DPO 2024), and maximal responses were measured from the traces following both direct and indirect stimulation. To normalize measurements by muscle size, the force generated during stimulation was divided by the estimated cross-sectional area of the muscle, which was calculated by measuring the diameter of the mid-belly of the muscle and calculating area assuming a cylindrical shape of the muscle. Synaptic efficiency was estimated as the ratio between indirect stimulation and direct stimulation for twitch and tetanic force.

### Vital imaging

Vital imaging experiments to repeatedly image individual NMJs in vivo, made possible by the fact that each NMJ has a unique pattern of nicotinic acetylcholine receptor (AChR) clusters that can be relocated, were adapted from the original methodology [24]. Vital imaging of the sternomastoid muscle (STM) requires that the breathing of the mouse, which causes movement of the muscle, be halted at the moment of image capture. To do so, the mouse must first be intubated for the purpose of mechanical ventilation, which necessitates injectable anesthesia. The animals were anesthetized with a ketamine/xylazine cocktail (65 mg/kg body weight ketamine, 5 mg/kg body weight xylazine) and placed on a stage in a supine position. Depilatory cream was used to remove the fur on the ventral side of the neck, an antiseptic iodine solution was applied to the skin, and a midline incision was made from the mandible to the sternum. An endotracheal tube was inserted into the mouse’s trachea and connected to a ventilator (Harvard Instruments, Model 683). The submandibular glands were pulled aside and the sternomastoid (STM) muscle was exposed using blunt dissection. The exposed STM muscle was bathed with 2 μg/mL BTX-488 (Alexa Fluor 488-tagged α-bungarotoxin, Invitrogen B13422) dissolved in sterile lactated Ringer’s for 5 min to allow for the visualization of the post-synaptic acetylcholine receptors. This BTX stain blocks acetylcholine receptors at a level far below that which is necessary to block transmission. Any excess BTX-488 was removed by several washes with lactated Ringer’s solution and the STM was slightly elevated using a small metal ring attached to a micromanipulator. No coverslips were used in these in vivo experiments. The neuromuscular junctions (NMJs) of the STM were then imaged using a Photometrics Cool SNAP HQ camera mounted on an Olympus BX51WI epifluorescence scope with a × 20, 0.95 numerical aperture water immersion objective. The light source used for epifluorescent imaging was an Olympus 100 W Mercury Power Supply (BH2-RFL-T3) with two neutral density filters of 50% and 25%. Images of slightly different focal planes were stitched together using the Photomerge Faces feature of Photoshop Elements 6 to create one image. This was done because NMJs are rarely entirely flat and *en face*, and therefore capturing slightly different focal planes was often necessary to image the full territory of an NMJ.

### Cardiotoxin application

Cardiotoxin (CTX) was applied by bathing the STM using a previously established methodology [9]. Animals were anesthetized with 4% isoflurane in an incubation chamber and then maintained on 1.5% isoflurane at 1 L/min oxygen. For these experiments, intubation was not required, so isoflurane anesthesia was used because it is easier to maintain a consistent surgical plane of anesthesia compared to injectables. The STM was exposed in the same way as for the vital imaging experiments and was bathed in 10 μM cardiotoxin isolated from the venom of *Naja mossambica mossambica* (Sigma C9759) dissolved in sterile lactated Ringer’s for 5 min. The wound was then washed numerous times to remove excess CTX, the skin sutured shut, and the animal was sacrificed at either 1 day or 7 days post-injury for observation of muscle regeneration.

### Statistics

Muscle force recordings were compared between groups using *t* tests with a Welch’s correction (Prism 7.0, Graphpad Software, California, USA). Statistical significance was considered to be a *p* value less than 0.05. All values are reported as mean ± standard error of the mean and were graphed using Prism.

## Results

### Myonuclei-specific transgenic fluorescent labeling in chemically fixed muscle preparations

**Fig. 1:**
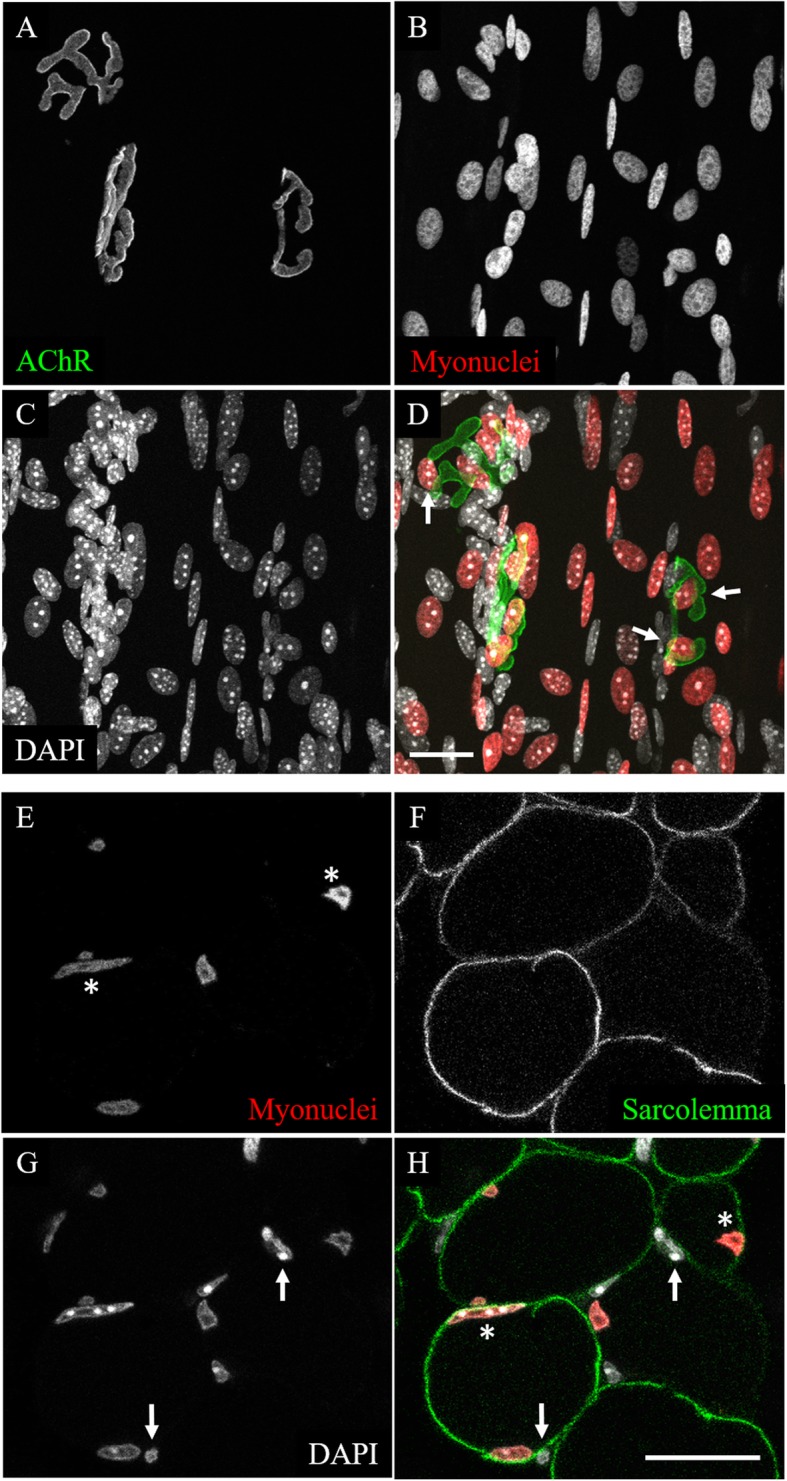
Confocal images of young adult RG STM muscles. **a-d** A maximum intensity projection of a whole-mount preparation. **a** BTX stain reveals AChR aggregates of three NMJs. **b** Myonuclei exclusively labeled with transgenic mCherry. **c** DAPI labeling of all nuclei in muscle tissue. **d** Composite image. Arrows indicate examples of myonuclei lying adjacent to, but not directly underneath, the AChR aggregates. **e-h** Single plane confocal image of a cross-section. **e** Myonuclei exclusively labeled with transgenic mCherry. Asterisks indicate examples of peripheral myonuclei. **f** Sarcolemmas stained with anti-dystrophin antibody reveal the boundaries of myofibers. **g** DAPI labeling of all nuclei in muscle tissue. Arrows indicate DAPI-labeled nuclei outside of the sarcolemma stain. **h** Composite image. Asterisks and arrows correspond to those in **e** and **g**. Scale bars 20 μm

The HSA-Cre79 transgene used in the RG mice is highly specific for myofibers in skeletal muscle tissue, based off our own observations presented in this paper and experiments performed by the group that developed the transgenic line [19, 25]. First, we wanted to ensure that mCherry signal in these mice would not be diminished by chemical fixation, so we fixed muscles from RG mice in 4% PFA. After fixation, we prepared the muscles for either whole mounts or cross-sections. Whole mounts of skeletal muscle are informative because they give an* en face* view of the tissue with the individual cells in their native form. Cross-sections are useful because they allow for the analysis of the muscle fibers within a muscle as a whole, whereas whole mounts are limited to surface muscle fibers. Identification and analysis specifically of myonuclei from images of whole mounts and cross-sections are difficult because there are a vast number of cell types that exist in skeletal muscle tissue and traditional nuclear stains, such as DAPI, do not identify nuclei of any particular cell type (Fig. 1c and g). The sheer amount of DAPI-stained nuclei obscures the myonuclei in the whole mounts by lying on top of or between the myofibers and in the cross-sections by being so close to the sarcolemma that it is often difficult to parse those nuclei within a myofiber versus those outside of it. When the mCherry label is imaged, however, fewer nuclei are labeled in the muscle tissue as a whole, as would be expected if only myonuclei are labeled (Fig. 1b and e). Interestingly, the subsynaptic nuclei seem to preferentially align adjacent to, rather than directly underneath, the BTX-stained AChRs (Fig. 1d). This specialized arrangement of subsynaptic myonuclei, clearly visible when AChRs (Fig. 1a) are viewed *en face* in whole mounts, is consistent across all RG NMJs we have observed. Figure 1e-h depicts an optical section within a representative 20 μm thick cross section of a young adult STM muscle. DAPI-labeled nuclei (Fig. 1g) are pressed close to the sarcolemma (Fig. 1f), either outside of or within the myofiber. Transgenically labeled myonuclei (Fig. 1e) are distinguishable from extra-muscular DAPI-labeled nuclei, and lie underneath the sarcolemma at the periphery of the myofiber. These results show that RG mice allow for the selective imaging of myonuclei in both whole mount and cross section preparations.

**Fig. 2:**
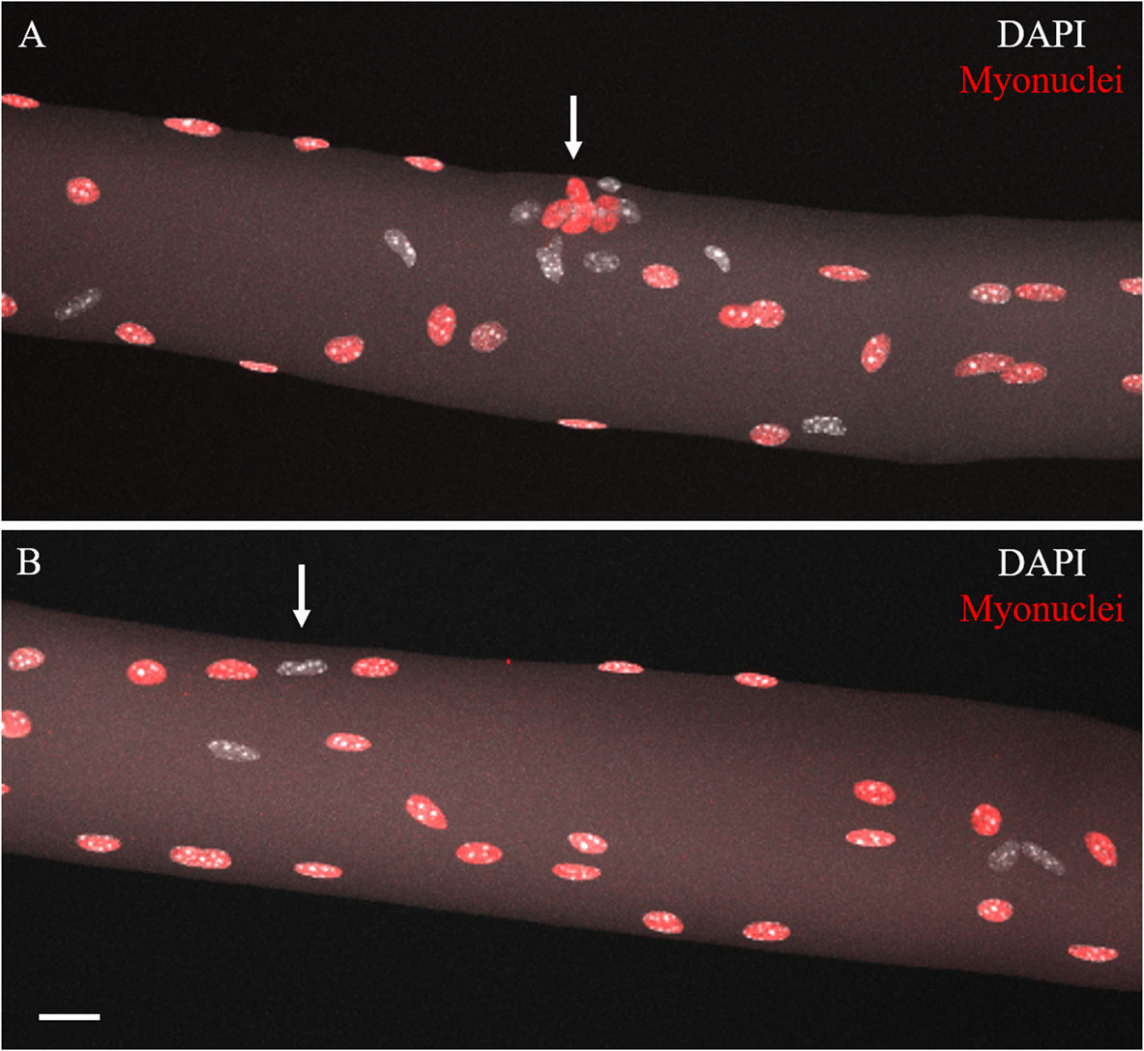
Maximum intensity projection confocal images of isolated single myofibers of young adult RG EDL muscle. Outlines of the individual myofibers were imaged using background auto-fluorescence. **a** Image of the synaptic area of a myofiber. The subsynaptic myonuclei (arrow) are identified by their tight clustering near the surface of the myofiber. **b** Arrow points to an mCherry negative, DAPI-positive nucleus among three mCherry positive nuclei that all appear to be within the sarcolemma. Scale bar 20 μm

It is difficult to determine whether a given nucleus is within the sarcolemma, and so we isolated single myofibers to observe myonuclei without extra-myofiber nuclei obscuring the image. In this preparation, fixed muscles were exposed to NaOH and single myofibers were teased apart. This single fiber isolation process destroyed the native mCherry and GFP fluorescence, likely due to the high concentration of NaOH needed for isolation, so immunostaining using an anti-mCherry primary antibody was used to recover the mCherry signal. Figure 2a and b shows representative images of single myofibers in which the mCherry fluorescence was recovered with immunostaining. Based on the boundaries of muscle fibers imaged with background fluorescence of the tissue, the anti-mCherry stained nuclei appear to reside within the fiber. There are, however, some nuclei (6.3 ± 0.6%, *n* = 5 single fibers) that do not have anti-mCherry stain but appear to reside within, or possibly pressed tightly to the surface of, the sarcolemma (arrow, Fig. 2b). The loss of native red fluorescence from exposure to reagents (including NaOH) during single fiber isolation indicates the denaturation of the mCherry protein. It is possible that such denaturation rendered mCherry unrecognizable by the anti-mCherry antibody in a subpopulation of myonuclei.

### RG mice exhibit normal muscle physiology

**Fig. 3:**
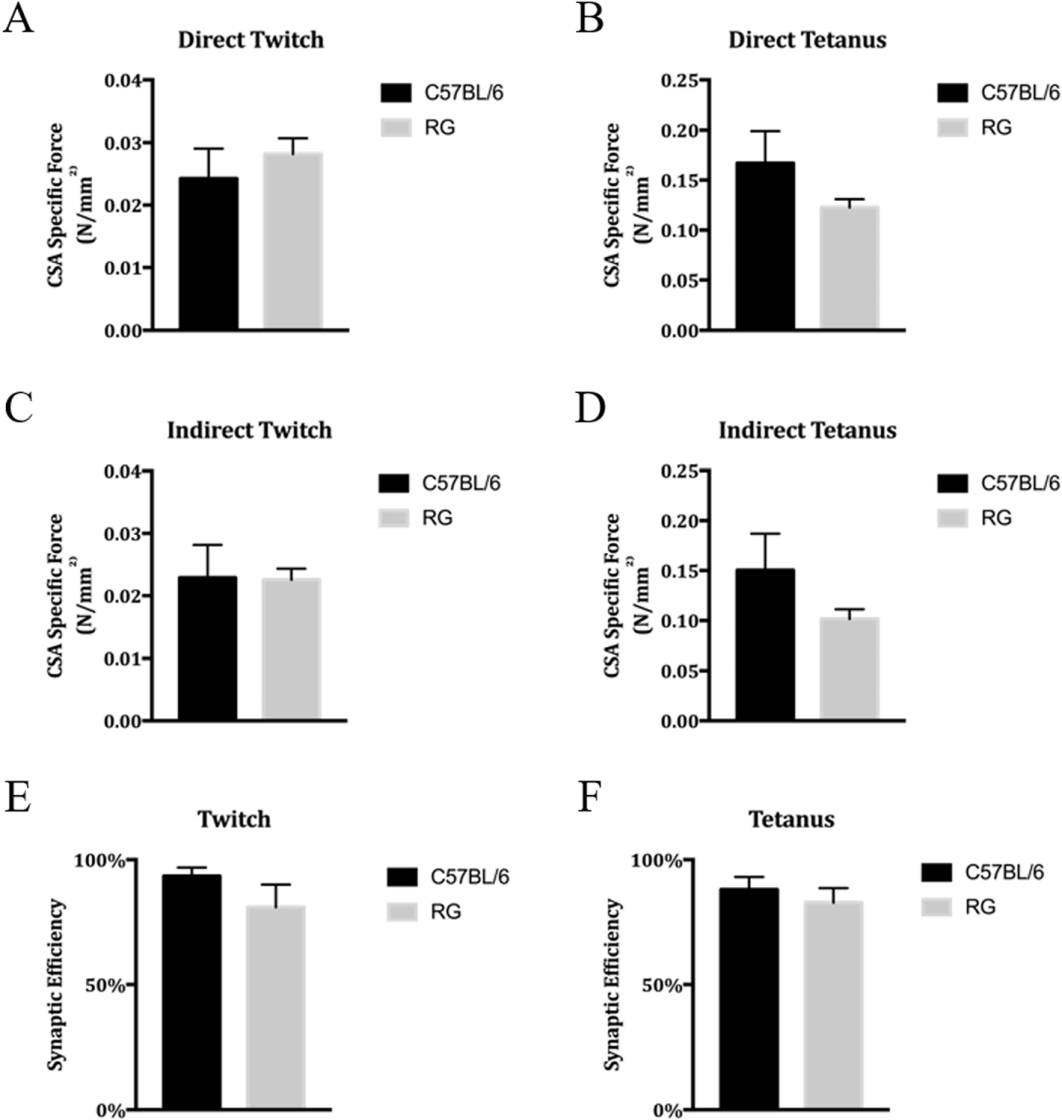
RG mice exhibit normal muscle physiology compared to C57BL/6 WT mice. All measurements shown here were normalized to the estimated cross-sectional area of each muscle. **a**, **b** Measurements of direct stimulation of muscle fibers. **c**, **d** Measurements of muscle twitch after nerve stimulation. **e**, **f** Estimates of synaptic efficiency using the data from direct and indirect stimulation. *n* = 3 age and sex-matched mice per group. There was no significant difference (as defined as *p* < 0.05) between any RG and C57BL/6 groups

In order to assess if mCherry expression in myonuclei or GFP expression in the sarcolemma affected the physiological function of skeletal muscles, the isometric contractile characteristics of the soleus muscle were examined (Fig. 3). Age and sex-matched C57BL/6 mice were purchased from Jackson Lab, acclimated to our mouse colony for several weeks, and then used as wild-type (WT) controls. There was no significant difference detected between WT and RG mice in any of the functional parameters measured. Each measurement was normalized to the estimated cross-sectional area of the muscle. The normalized evoked twitch and tetanus maximum force generation did not differ between RG and WT animals, stimulated either directly (Fig. 3a and b) or indirectly through the nerve (Fig. 3c and d).

To assess the function of the neuromuscular junction, a ratio between the indirect and direct twitch responses was used to determine the efficiency of neuromuscular transmission from nerve to muscle. Theoretically, if the synapse is 100% efficient, the direct and indirect twitch responses would be equal, though this is rarely the case, even in healthy synapses. Once again, the synaptic efficiency of WT compared to RG muscle was not statistically different. In all, we determined that the transgenic fluorescent protein tags of the sarcolemma and myonuclei do not result in the impairment of skeletal muscle physiology.

### RG mouse enables prolonged, time-lapse in vivo imaging of myonuclei

**Fig. 4:**
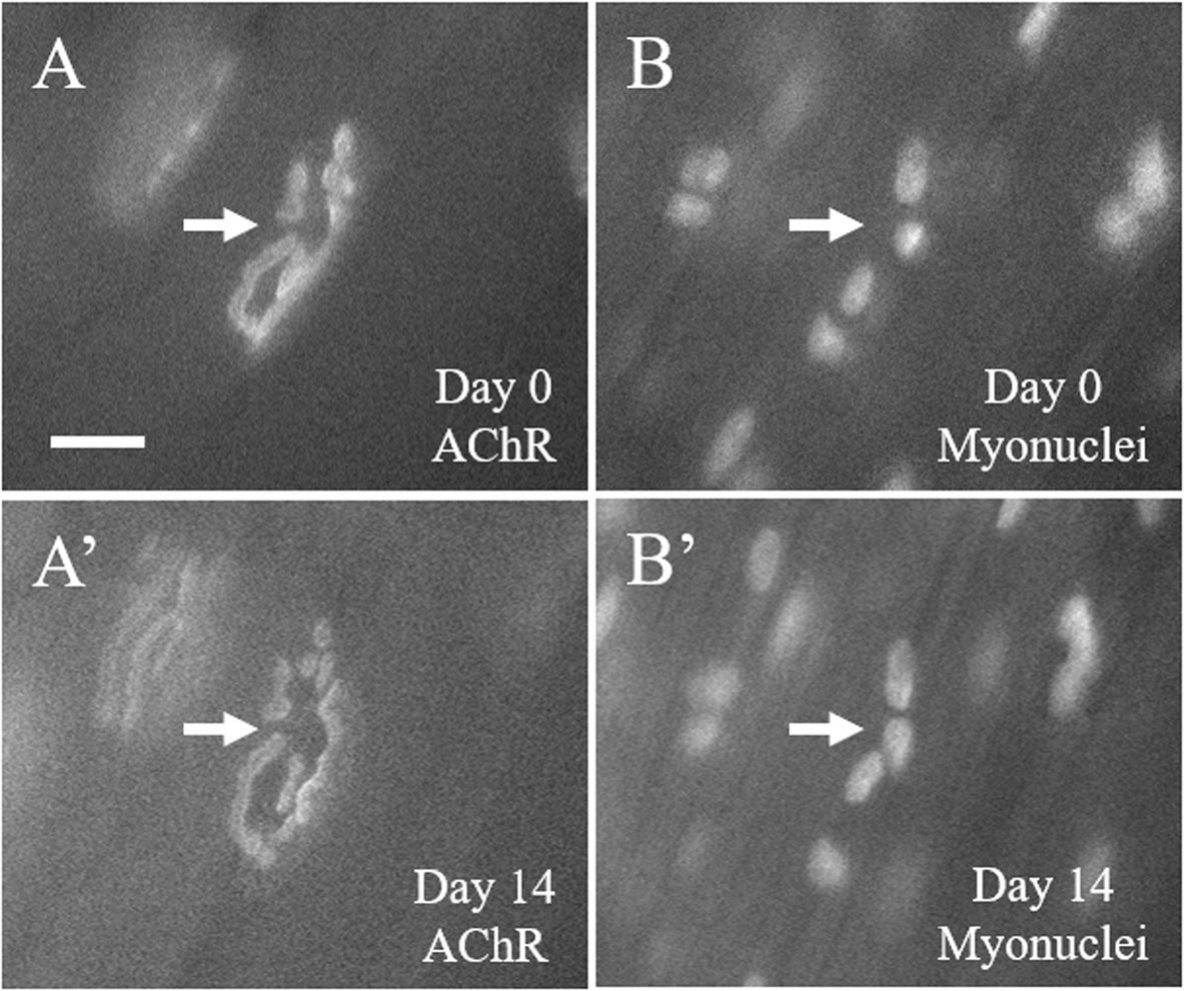
Vital images of the same STM NMJ from a young adult RG mouse 2 weeks apart. **a**, **a’** Epifluorescent images of AChRs labeled with fluorescent BTX. **b**, **b’** Epifluorescent images of myonuclei labeled with mCherry-tagged histone H2B protein. **a**, **b** Images of an NMJ during the first imaging session. **a’**, **b’** Images of the same NMJ 2 weeks later. The arrows point to a single NMJ and its corresponding four subsynaptic nuclei. The subsynaptic nuclei are clearly visible in both **b** and **b’**, and do not appear to have undergone major positional changes. The slight differences in the appearance of the AChRs and myonuclei between the two imaging sessions can likely be accounted for by a slightly different stretching of the muscle in each session. Scale bar 20 μm

A major methodological advance enabled by the RG mice lies in the ability to repeatedly image myonuclei in the living animal. Using the technique of vital imaging of NMJs, we have repeatedly imaged several NMJs in multiple mice. Figure 4 shows a representative NMJ, imaged twice with 2 weeks in between each session. While vital imaging the entire length of a myofiber is impractical, the junctional area, and in particular the subsynaptic nuclei, which are transcriptionally distinct and provide the transcripts necessary to maintain the NMJ [26, 27], are readily imaged. Furthermore, because this label is transgenic, it allowed us to repeatedly image myonuclei of the same NMJ, identified by its unique pattern of AChR clusters. Between the two imaging sessions of the individual NMJ shown in Fig. 4, there appear to be no major changes to either the AChRs or the subsynaptic nuclei.

**Fig. 5:**
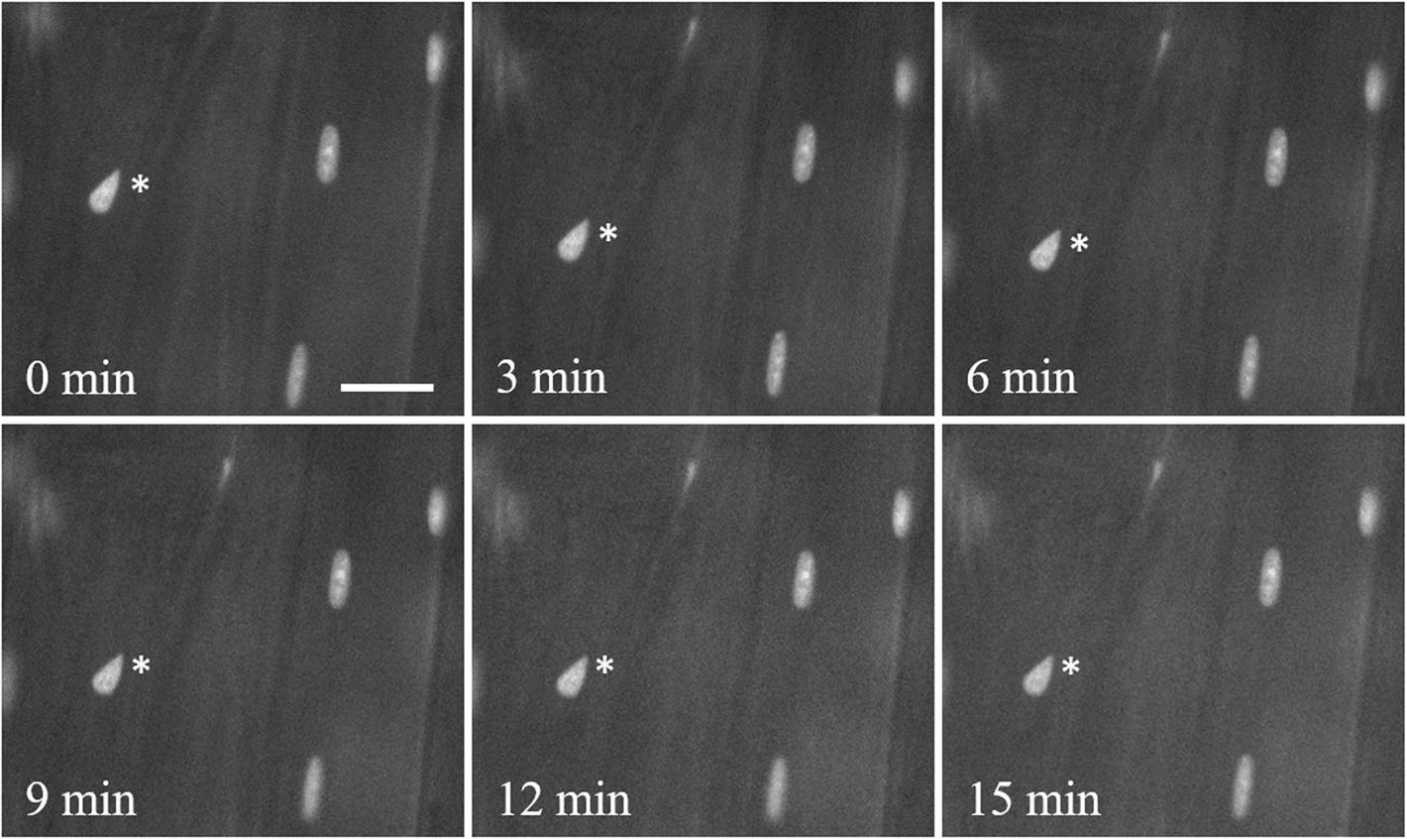
Prolonged vital imaging of nuclear label. The same set of nuclei were repeatedly imaged in vivo every 3 min over the course of constant exposure to green excitation light for 15 min. Asterisks indicate the same myonucleus at each time point. No change in the ability to image the nuclei was detected. Scale bar 20 μm

In order to verify that the mCherry label of myonuclei was sufficiently stable to image over prolonged periods of time, we continuously illuminated a single spot in a muscle for 15 min using an epifluorescent TRITC filter cube set. Such continuous illumination was performed in three mice and the same myonuclei within the fields of view were imaged every 3 min. In each case, the nuclei produced the same quality image without having to adjust any imaging parameters during the experiment. Figure 5 shows representative images from one mouse. These results show that the RG mice provide a myonuclear label that is sufficiently stable to image for extended periods of time.

### RG mouse permits tracking of myonuclear disposition in various experimental paradigms

In order for RG mice to be useful in the study of myonuclei, the myonuclear label must be present and robust not only in normal muscle tissue but also in muscles that deviate from the healthy, young phenotype. To determine whether central myonuclei were transgenically labeled in models of skeletal muscle damage and repair, we examined RG muscles exposed to a myotoxic compound (cardiotoxin), muscles of *mdx* (dystrophic) animals carrying the RG construct, and aged RG mice.

**Fig. 6:**
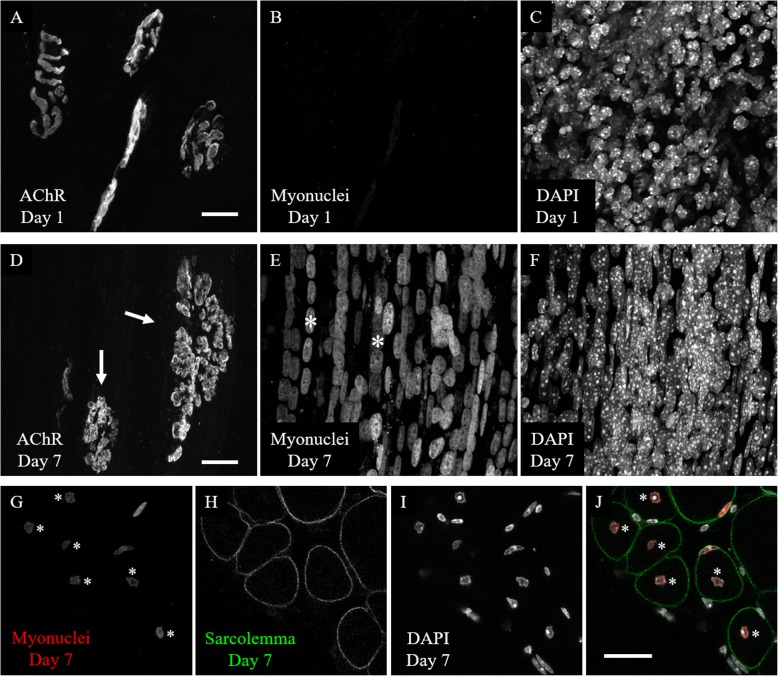
Confocal images of cardiotoxin-damaged STM muscles from young adult RG mice. **a-c** A maximum intensity projection of whole mounted muscle fibers one day post-CTX injury. **a** AChRs largely retain their uninjured morphology. b The mCherry labeled myonuclei are entirely absent from the myofibers 1 day post-myofiber damage. **c** An abundance of DAPI-stained nuclei resides within the muscle tissue. **d**-**f** Whole-mounted muscle fibers 7 days post-CTX injury. **d** AChRs 7 days after injury have taken on the fragmented phenotype (arrows) common to regenerated myofibers. **e** Seven days after injury, mCherry labeled myonuclei re-appear in long strings located in the center of the myofibers. Asterisks indicate examples of strings of central myonuclei. **f** The numerous strings of central myonuclei stain with DAPI. **g**-**j** Single plane confocal image of a 20 μm cross-section of muscle fibers 7 days post-CTX injury. **g** Transgenic myonuclear label. Asterisks indicate centrally positioned myonuclei. **h** Sarcolemmas stained with anti-dystrophin antibody reveal the boundaries of myofibers. **i** DAPI labeling of all nuclei in muscle tissue. **j** Composite image. Asterisks correspond to those in **g**. Scale bars 20 μm

To test whether the RG mice would be useful in the study of central myonuclei resulting from acute myofiber injury, cardiotoxin (CTX) was used to damage the STM muscle of young adult RG mice. Mice were sacrificed 1 day or 7 days after the injury. In each mouse, the STM was damaged by bathing the muscle in 10 μM cardiotoxin dissolved in sterile lactated Ringer’s for 5 min. This procedure damaged a large number of fibers on the exposed surface of the muscles, but largely spared interior muscle fibers and the fibers on the underside of the muscle. Figure 6 shows representative images of muscles from cardiotoxin experiments using the RG mice. The mCherry label of myonuclei was lost upon the degeneration of myofibers 1 day post-injury (Fig. 6b), but re-appeared when the myonuclei returned in strings of central nuclei 7 days post-injury (Fig. 6e). Myofibers regenerate, with prominent central nuclei, 6 to 7 days after damage [7] and the RG mice at 7 days post-injury display strongly labeled myonuclei. AChRs still appear in their normal “pretzel”-like configuration 1 day after injury (Fig. 6a), although the stain appears to be faint in some areas. It is possible that the fluorescent BTX at this time point labels AChRs that are stuck in the basal lamina after degeneration of the myofiber, as it has been previously observed that myotubes plated on laminin leave behind AChRs affixed to the substrate after mechanical removal of the myotubes [28]. The affected areas of the muscle are covered in a large number of DAPI-stained nuclei (Fig. 6c), which may be immune cells invading the tissue 1 day post-injury. At 7 days, the AChRs of the affected area have become fragmented (Fig. 6d) and many strings of DAPI-stained central myonuclei are present (Fig. 6f). Figure 6g-j depicts a cross-sectional view of a STM muscle 7 days after cardiotoxin injury. In cross-section, the mCherry labeled myonuclei (Fig. 6g) are shown to reside within the center of the sarcolemma stain (Fig. 6h). These observations are consistent with well-established phenotypes after muscle injury and, importantly, mCherry signal returns concurrently with the formation of new myofibers, so the RG mice will be useful for studying myonuclei after injury.

**Fig. 7:**
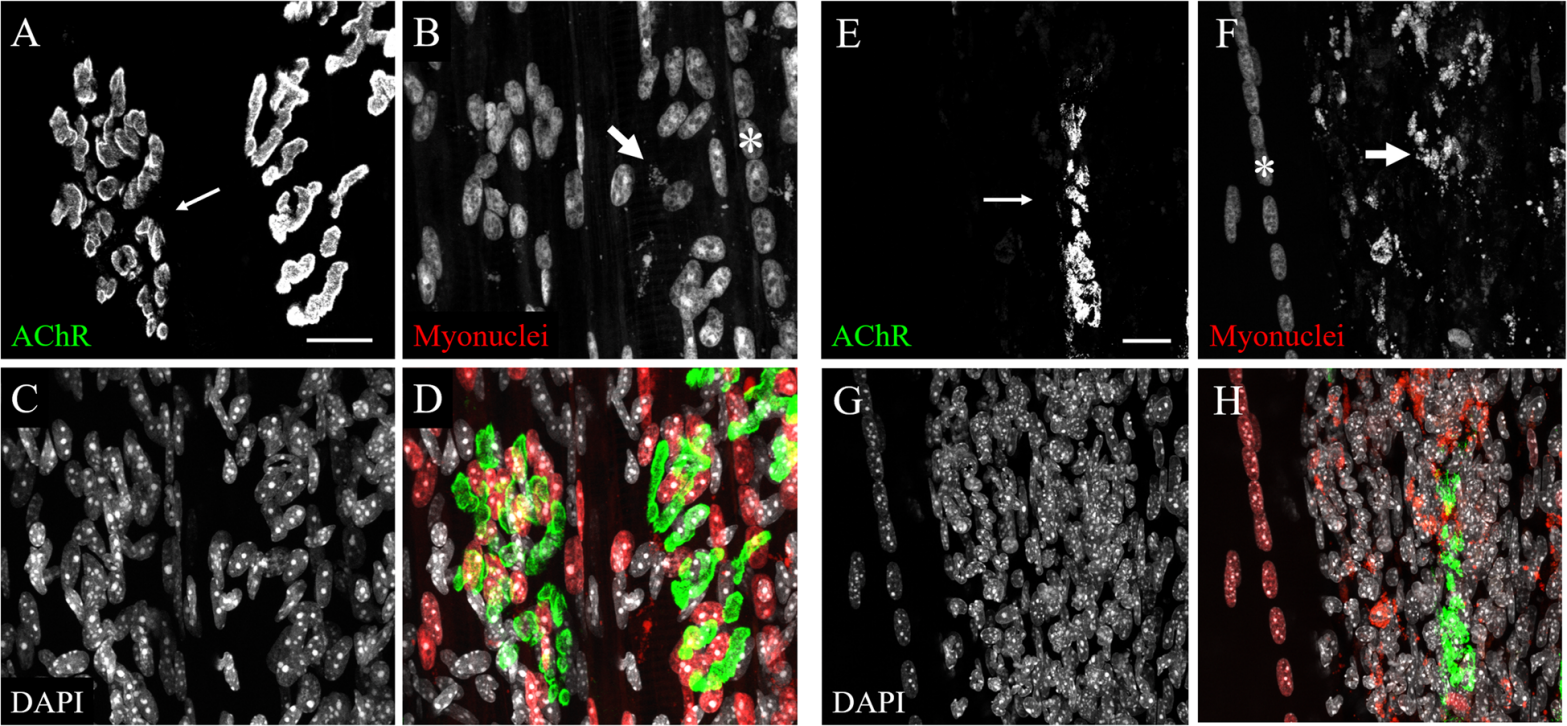
Maximum intensity projection confocal images of whole-mounted RG/*mdx* SOL muscle. **a** and **e** AChRs stained with BTX, which have a fragmented appearance (thin arrows) typical of *mdx* NMJs. **b** and **f** Transgenic mCherry label of myonuclei. There is a string of central myonuclei (asterisk) and sparse, scattered mCherry signal (thick arrow) in each panel. **c** and **g** DAPI-stained nuclei. Note that adjacent areas of the muscle tissue have drastically different amounts of various nuclei within the tissue in panel **g**. **d** Composite image of **a**-**c**. **h** Composite image of **e**-**g**. The mouse used for these images was homozygous for the H2B/GPI reporter allele. Scale bars 20 μm

We next sought to explore whether the RG mice could be used to visualize the myonuclei of mouse models of myopathies. The *mdx* mouse is a widely used murine model of Duchenne muscular dystrophy: both *mdx* mice and DMD human patients harbor mutations in the same causative gene which codes for dystrophin protein [22]. Skeletal muscle fibers of *mdx* mice have a preponderance of central nuclei, so we sought to use the RG transgenic label to visualize central myonuclei in the *mdx* model. To this end, the HSA-Cre79, H2B/GPI, and *mdx* alleles were bred into the same mouse lineage, to create the RG/*mdx* line. Two mice from this line were sacrificed, and representative images of whole-mounted soleus (SOL) muscle tissue are shown in Fig. 7. The AChR aggregates appear dispersed and fragmented (Fig. 7a and e), as expected in dystrophic muscle. The myonuclei are well labeled with the transgenic mCherry in the RG/*mdx* line (Fig. 7b and f), and strings of central myonuclei are clearly present (asterisks, Fig. 7b and f). In this group of images, there are myofibers that are covered in DAPI-stained nuclei (Fig. 7c and g), and many of the nuclei in this area are surrounded by a dispersed, granular mCherry signal (thick arrows, Fig. 7b and f). These may be remnants of fluorescently labeled myonuclei still being digested within phagocytic cells that accumulate in response to muscle injury (see the “Discussion” section). In Fig. 7g, there is an area with dense DAPI stain (Fig. 7f, right side) adjacent to a myofiber surrounded by relatively few DAPI-stained nuclei but with a long chain of central myonuclei (asterisk, Fig. 7f), which is presumably a myofiber that has recently undergone regeneration.

**Fig. 8:**
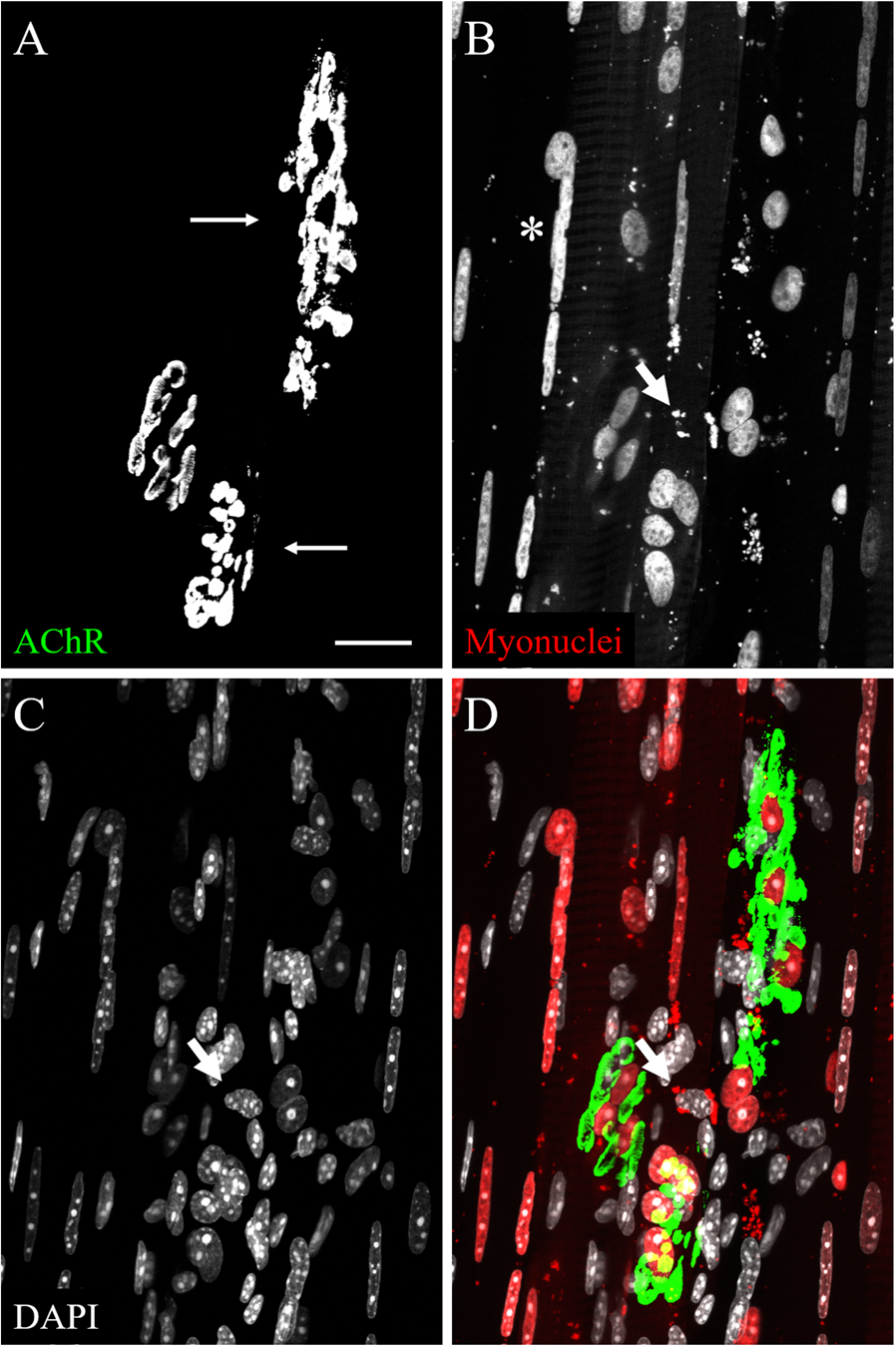
Maximum intensity projection confocal images of whole mounted aged RG STM. **a** AChRs stained with BTX, which have a fragmented appearance typical of aged NMJs (thin arrows). **b** Transgenic mCherry label of myonuclei. There is a string of central myonuclei (asterisk) and sparse, scattered mCherry signal (thick arrow). **c** DAPI stain of all nuclei in the tissue. **d** Composite image of **a-c**. Scale bar 20 μm

As mice age, their skeletal muscle tissue tends to develop some characteristics in common with *mdx* mice and intentionally damaged myofibers, including more numerous instances of fragmented endplates and central myonuclei [15, 16]. To test whether the mCherry label could be used to study myonuclei in the muscles of old mice, RG mice were aged in our vivarium. Three aged mice (19-22 months old) were sacrificed and their muscles harvested. Figure 6 shows representative images from the STM of a 22 month old RG STM muscle. The muscles of this aged RG mouse show numerous myofibers containing central nuclei that are well labeled with mCherry (asterisk, Fig. 8b). Similar to the RG-*mdx* mice, there is a sparse mCherry label that often surrounds a DAPI-stained nucleus that is not itself labeled with mCherry (thick arrow, Fig. 8b).

## Discussion

Here, we have shown that the RG mouse line provides an excellent tool to identify and follow myonuclei in skeletal muscle injury, disease, and aging, in both living and fixed tissue. The fluorescent label of myonuclei is robust in normal skeletal muscle tissue, recovers after myofiber damage, endures in repeated high damage scenarios, such as the *mdx* mouse, and persists through old age of the animals. This tool can be extended to study the myonuclear dynamics of models for myopathies, aging, and muscle damage.

To our knowledge, the only other method to label myonuclei of entire myofibers in the living mouse to date has been to insert fluorescent-tagged oligonucleotides into individual myofibers via injection or electroporation [29–31]. This method labels the nuclei of single myofibers without labeling the rest of the muscle, which can be advantageous, but necessitates damage to the sarcolemma in order to introduce fluorescently labeled oligonucleotides and is incompatible with experiments that require the labeling of myonuclei in all muscle fibers of any given muscle. The RG mice require no manipulation of the myofiber to image the nuclei.

This Cre-inducible reporter construct was designed to produce a GFP fluorescent tag of the plasma membrane. It was our hope that the GFP-tagged GPI membrane insertion sequence would create a fluorescent label of the sarcolemma that would allow us to determine whether nuclei lie within the sarcolemma, but the membrane-bound GFP signal in the RG animals proved to be unreliable in sarcolemmas, often below the threshold of detection or with non-uniform surface distributions, and there was often GFP signal detected within the nuclei. Therefore, the native transgenic GFP label was not useful for our purposes. We attempted to enhance the GFP signal with two different anti-GFP antibodies (Life Technologies A6455 and Aves GFP-1020), neither of which produced an appreciable signal. It is unclear why the GFP signal is so weak under the control of this HSA-Cre79 transgene while other Cre transgenes produce excellent labeling of cell membranes [20].

There are numerous questions regarding myonuclei that can be investigated using this vital label. For example, the consequences of central myonuclei on the function of muscle fibers are not clear [32]. It is possible that central nucleation has a deleterious effect on the contraction of the myofiber. The RG mice can be used to take direct physiological recordings from a myofiber with central nuclei. It is also unclear why the strings of central nuclei are of different lengths in different myofibers. The RG mice could be used to survey numerous myofibers in whole-mounted tissue to determine the lengths of various strings of central nuclei. There are also questions about whether and when central nuclei migrate back to the periphery of the myofiber after damage that can be answered using a vital label of myonuclei. It has been reported that myonuclei migrate to the periphery within 8 days if only the central third of the myofibers are crushed [7], but others have reported that central nuclei persist for longer than 3 weeks after cardiotoxin injury, and even that they are a permanent fixture of a myofiber having endured such insult [8]. It is possible that there are differences in rates of myonuclear migration in relation to the severity of damage inflicted on a muscle, and the RG mice could be used to conduct definitive experiments exploring this question.

The characterization of the mCherry label in RG muscles has already yielded interesting observations. For example, the subsynaptic nuclei seem to preferentially align adjacent to, rather than directly underneath, the BTX-stained AChRs. An explanation for this specialized arrangement of subsynaptic myonuclei could be that protein synthesis machinery that surrounds the nucleus, such as endoplasmic reticulum, is aligned directly under the synaptic gutters, leaving the nucleus to be positioned slightly to the side. Also, a feature common in all models of abnormal skeletal muscle tissue presented here (intentional damage, *mdx*, aging) is the increased presence of DAPI-stained nuclei that lie outside of the muscle fibers. In the aged and *mdx* animals, these areas of immense extra-myofiber nuclei are accompanied by mCherry signal that is dispersed and granular, often surrounding DAPI-stained nuclei. Considering that chronic muscle damage and inflammation are common in aged and *mdx* mice, it is possible that the DAPI nuclei belong to immune cells that phagocytose myonuclei from the damaged area of the muscle tissue, thereby producing DAPI stain surrounded with mCherry signal. Furthermore, the fact that at 1 day post-injury of young, healthy muscle tissue no mCherry label was left or observed in a dispersed, granular form suggests that the myonuclei of the damaged fibers may have been phagocytosed in a faster and more efficient manner than in the aged and *mdx* muscles. The RG mice may be useful for the in vivo observation and comparison of myonuclear degradation and the clearing of damaged muscle tissue in young, healthy animals compared to old or myopathic animals.

## Conclusions

The role and consequences of myonuclear disposition and dynamics on the development, function, and regeneration of myofibers is an understudied, yet potentially important field of skeletal muscle research. The ability to specifically investigate myonuclei is limited, however, by the lack of reliable vital labels for myonuclei. The mouse line described here, with a transgenically expressed myonuclear label, provides a superior tool to study myonuclei in healthy, damaged, diseased, and aged skeletal muscle fibers in living and fixed tissue.

## Data Availability

All data presented in this article are available from the corresponding author upon reasonable request.

## Abbreviations

NMJ: Neuromuscular junction
STM: Sternomastoid
SOL: Soleus
EDL: Extensor digitorum longus
BTX: α-bungarotoxin
AChR: Acetylcholine receptors
PBS: Phosphate buffered saline
PFA: Paraformaldehyde
CTX: Cardiotoxin
HSA: Human α-skeletal actin
GPI: Glycosylphosphatidylinositol
GFP: Enhanced green fluorescent protein
DAPI: 4′,6-diamidino-2-phenylindole
